# Case of Cancer, Illustrative of the Treatment by Pressure

**Published:** 1817-05

**Authors:** George T. Dunning

**Affiliations:** Surgeon.


*For the London Medical and Physical Journal.*

*Case of Cancer, illustrative of the Treatment by Pressure;* by George T. Dunning, Esq. Surgeon. Communicated by William Woollcombe, M.D.

MRS. FOOT, ætat. 59, consulted us early in January 1816, for a tumor in her left breast. She was a very tall and large-boned woman; her hair light, and her complexion rather fair. Until the commencement of the disease in her breast, she had enjoyed uninterrupted good health, and her strength was greater than that of almost any other woman. We observed, however, on one side of her neck, near the angle of the jaw, some deep and extensive cicatrices,—the consequence, she was told, of glandular suppurations when about six years of age. She was the mother of several healthy children, all of whom she suckled, and her catamenia had left her between seven and eight years.

About

About fourteen months before she applied to us, she discovered, for the first time, a hardness in her left breast, which was then attended with little or no pain. As it increased in size it became also more troublesome to her, and she had lately suffered the severest lancinating pains, darting backwards from the breast to the shoulder-blade, and occasionally down the arm. The tumor, when I first saw it, was about the size of a small egg, of a stony hardness and nodulated figure, and was imbedded in the glandular structure of the breast. The superficial veins of the breast were generally turgid, and the integument immediately covering the tumor (though there had never been a wound there) had the appearance of an old cicatrix. It had recently ulcerated in one point to about the extent of a kidney-bean, and the discharge from it, which was thin and in considerable quantity, was attended with that peculiarly offensive and characteristic fætor, which cannot, I believe, be mistaken. There was apparently no attachment of the tumor to the subjacent muscle, nor could we discover any glandular enlargement in the axilla, or any knottiness or trace of lymphatic disease between it and the breast; yet I may venture to assert, that every feature of this case was unequivocally that of the most decided and genuine scirrhus. Her bowels had for a long time been very irregular; her appetite bad; she was much emaciated; had a dry, short, and petulant cough, with night perspirations, and appeared to be decidedly hectical. Looking to the insulated state of the disease, and, at the same time, to the deranged action of the stomach and bowels, we flattered ourselves, that if, in a short time, by correcting this, we could improve a little her general health, we might then have recourse to the removal of the breast with some prospect of success. About the middle of February, though the ulcer had extended a good deal, and its surface was very foul and sloughy, yet, as we had succeeded in a great degree in our views relative to her general health, we were about to propose to her the operation. At this time, however, the perusal of Mr. Young's Treatise on Cancer hardly left us an alternative. We did not feel justified in withholding a trial of the novel and apparently successful mode of treatment, which, after considerable experience, he had recommended, and which was indeed exciting great interest every where among medical men.

February 18, 1816.—The ulcer being filled with finely powdered chalk, adhesive straps were applied over the tumor; next to these, several thick compresses of linen; and the whole was secured by a long flannel bandage, which was put on with considerable firmness. She spoke of little pain from

 it, it, and, on the whole, passed the three or four succeeding days with more comfort than usual. The lancinating pains returned occasionally, but with less frequency and violence.

22d.—The dressings were now removed for the first time. Neither the tumor or ulcer appeared to have undergone any material change. The sanious discharge was equally offensive and copious, her cough rather more troublesome. The dressings were replaced with an additional compress of linen, and, between the linen compresses, a piece of thin lead once or twice doubled.

25th.—She had felt little or no pain in the affected part since the application of the lead. On exposing the breast to-day, the whole volume of it seemed considerably reduced, the tumor was both smaller and flatter, the sanious discharge was less copious and considerably less offensive; the wound was more superficial, its edges excoriated, and its surface generally sloughy; but, on one side of it, we observed with great satisfaction a point or two of granulation. The excoriated parts being covered with gold-beater's skin, the compresses and lead were replaced, and the bandage applied with a degree of pressure that was almost extreme,—at least, we were much surprised that she could bear it. She was directed to take, every night, Hydrarg. Submur. gr. ifs. et Ext. Conii. grs. v.

March 2d.—The tumor was still lessening, the ulcer in one part filling up with florid healthy granulations, the discharge inconsiderable in quantity, and scarcely at all offensive. Her cough, however, having been more troublesome for the last day or two, Opii. gr. fs. et Pulv. Scillæ. gr. i. were added to her night-pill, and she was ordered to take occasionally a spoonful of an emulsion with Tinct. Hyoscyami.

8th.—The breast had for several days been regularly mending, but to-day it took on again a much less favorable complexion. Its edges looked irritable and ragged, and many of the granulations had become absorbed. It should be remarked, that her cough, during the last day or two, harassed her exceedingly. Her gums having become a little sore, the calomel in the night-pill was omitted, and the opium increased to a full grain.

12th.—The breast appeared to-day at all points worse; the wound was both broader and deeper, its surface exceedingly foul, the discharge as offensive as ever, and the pain intense. Such was the violence of her cough, and such necessarily the jerking motion of the chest in the act of coughing, that the breast, begirt as it was and compressed by the bandage and plates of lead, was literally in a state of almost constant percussion,—and to this circumstance we attributed, in a great degree, as also did our patient, the present unfavorable state of things. The bandage was now applied less tightly.

16th.—The breast would no longer bear even the gentlest pressure, so that we were compelled to lay aside our bandage, and to dress it simply with dry lint; it was also bathed often with a decoction of camomile flowers in milk. Her cough was incessant, she had sweat a good deal during the last few nights, and had lost all appetite.

21st.—The pain having materially abated, though still very considerable, and her cough being much relieved by a free expectoration of mucus, we were again induced to have recourse to gentle pressure. Linen compresses only were used, without the plates of lead; and, instead of filling the wound with chalk, as was done before, we dressed it simply with an ointment of Ung. Ceræ. et Pulv. Opii.

28th.—Her cough having been much relieved during the last three or four days, and the pain so slight as to admit the application of firm pressure to the breast, every thing again looked well. The process of granulation was again advancing rapidly; there was scarcely any remaining hardness of the breast; and the discharge, which was almost intolerable from its fœtor, had now become a bland "laudable pus." Dr. Woollcombe, who, I regret, did not see her when the disease was at its worst, visited her with us this day, and was equally surprised and pleased, on learning from us the preceding circumstances of the case, at the present very favorable and promising state of it. Indeed, the conversion of a foul ulcerated scirrhus, by pressure only, into an apparently simple and granulating sore, would have been so much a matter of astonishment to ourselves, (if we had not been prepared to expect it by Mr. Young's pamphlet,) that, even with the fact before us, we should have almost doubted its reality.

April 6th.—The breast since the last report has been regularly progressive, but her cough was somewhat worse, in consequence, it would seem, of exposure to a severe easterly wind, which prevailed here about this time, occasioning much pneumonic disease. She was now desired, if possible, to do without her anodyne; her bowels, which from the commencement of our treatment had uniformly been kept lax, by an occasional dose of Epsom salts, or some other mild aperient, were now more actively opened, and a large blister applied between her shoulders.

10th.—Dr. Woollcombe saw her again this day, and was again much pleased with the progressive improvement of the breast since his former visit. There now remained no hardness of it, and the sore was not more than one-third its size when when we first applied the pressure. Her cough, however, though relieved by the blister, was still so exceedingly troublesome as to lead to an apprehension in Dr. W.'s mind, as well as in our own, that much serious mischief might be going on in the lungs. With this view, Dr. W. proposed that a seton should be made between her shoulders, but she could not be prevailed on to submit to it.

12th.—In consequence of our patient having incautiously ventured out of doors, (the cold easterly wind still prevailing,) we found her to-day laboring under complete pneumonia; the ultimate event of which, under the circumstances which have been already related, may be readily anticipated, and described therefore in a few words. Though the lancet was immediately used; though she was thrice bled as copiously as the state of her constitution would admit; though large blisters were applied to her chest, and the antiphlogistic and other means employed, which the nature of the case would suggest; the lungs could not be prevented from going into abscess. She fell irrecoverably into confirmed phthisis, under which she lingered till the beginning of June. Such was the extreme dyspnœa from the first attack of inflammation to her death, that the bandage, from its limiting the respiratory movements of the chest, was almost insupportable. In consequence of its being laid aside, and no doubt too of the general decay of the system, the reparative process which had been going on in the breast necessarily ceased. The wound soon became foul and ill-conditioned, without however making, I believe, any approach to the cancerous character, which had been so strongly and decidedly marked before the employment of the pressure.

Few surgeons would hesitate to place the preceding case in the scale of favourable reports; for, reviewing it in connexion with those brought forward by Mr. Young, in his late valuable work on Cancer, it cannot be denied, that pressure has a great controling influence over the cancerous principle; nor would it, I conceive, be a violence to probability to admit, that, if pneumonia had not supervened, and there had been less too of previous constitutional affection, the local disease might have been in this instance wholly and permanently removed. If, however, it should be determined by future experience, that we cannot always, by regulated pressure, supersede the action of cancer, we shall be able probably, in a large proportion of cases, to convert, or, if I may be allowed the expression, to bring it back to a suppurating point, and keep it there for a considerable time. Admitting the power of effecting this very important change, there is reasonable ground for hope that the surrounding healthy healthy parts, after the free removal of the breast under this state of things, will be much less liable to take on the late long-associated diseased action, than if the removal had taken place when that action existed in full force. It is obvious too, and not unimportant, that, while the cancerous action is thus suspended, general remedies may be employed, if the constitution requires them, for placing it in circumstances still more favorable for the operation. Such an opinion is unquestionably countenanced by some seeming analogies in the practice of surgery. Has it not hitherto been a maxim, when a lower extremity, for example, is in a state of sphacelus, to wait until the line of separation has taken place, or, in other words, until the action of sphacelus is arrested, before having recourse to amputation; and, where this rule has not been observed, has not the stump for the most part taken on the same diseased action which led to the removal of the limb? Analogies like these might, I believe, be multiplied; but the frequent recurrence of this afflicting disease after the operation, as it has been hitherto performed, will dispose medical men to pay the readiest attention even to a hint casually thrown out, which it is possible may ultimately lead to more successful results. After all, it would be uncandid and unprofessional were I to close this case without observing, that a question often arose in my mind, during the course of it—and it is a question, I believe, that will arise in the mind of every one who reads it—whether the quick absorption of so large a mass of diseased gland can be effected, (every particle of which, we have always thought, contained in it the leaven of the most destructive and malignant principle which the human body is capable of generating,) and the materies morbi carried off through the common emunctories of the body, without falling, in its passage through the system, on the lungs,* or liver, or stomach, or other parts more immediately vital than that from which it has been brought, and thus hasten, rather than retard, the fatal termination of this dreadful disease. Those who incline to the unfavorable side of the question, will refer the sudden increase of pulmonary affection in our late patient, and, it will follow, the acceleration of her death too,

* It is almost needless to observe, that, in the latter stages probably of most cases of cancer, when the vis medicatrix has ceased to oppose any longer the progress of it into the system, these viscera have been found studded with scirrhus tubercles. Many preparations of these are preserved in almost every collection of morbid anatomy. We regretted extremely that we could not obtain leave to ascertain the state of these viscera in our patient.

more more readily to the effects of absorbed disease, than of exposure to easterly winds, or to any other cause. But, on the other hand, it may not be improbable that the specific action or properties of cancerous virus may, by the agency of pressure, be at least much changed, if it be not destroyed, before such rapid absorption as we have just seen can take place; and, therefore, if happily it should hereafter be ascertained, that pressure is capable of producing effects like these, it may be presumed, that *(materie morbi mutatâ)* the quick and large absorption of it, under the substitution of a seton or issue some where, at the same time will be harmless, both in its immediate and remote effects on the constitution. That the cancerous principle in its unchanged state is not readily absorbable into the system, seems to be undeniably proved by the not infrequent instances of ulcerated scirrhus continuing for many months, now and then for some years, without producing any material constitutional derangement, or even extending to a great degree its local effects. And, indeed, a very few cases would warrant almost a belief, that these destroy at last more by profuse hæmorrhages and the effects of local ravages, than directly from a translation of the morbific principle into the constitution.

Mr. Young is much entitled to the thanks of society; he has called the attention of the medical world to new and important views of the most afflicting disease to which the human body is exposed.

*Plymouth Dock; March* 21, 1817.

